# Economic Burden of Hospitalizations for Heat-Related Illnesses in the United States, 2001–2010

**DOI:** 10.3390/ijerph13090894

**Published:** 2016-09-08

**Authors:** Michael T. Schmeltz, Elisaveta P. Petkova, Janet L. Gamble

**Affiliations:** 1 ASPPH/EPA Environmental Health Fellowship Program at the United States Environmental Protection Agency, Office of Research and Development, National Center for Environmental Assessment, Washington, DC 20001, USA; 2 National Center for Disaster Preparedness, Earth Institute, Columbia University, New York, NY 10027, USA; epp2109@cumc.columbia.edu; 3 United States Environmental Protection Agency, Office of Research and Development, National Center for Environmental Assessment, Washington, DC 20460, USA; gamble.janet@epa.gov

**Keywords:** climate change, health equity, heat-related illness, hospitalizations, economic cost, racial disparities

## Abstract

Understanding how heat waves affect morbidity and mortality, as well as the associated economic costs, is essential for characterizing the human health impacts of extreme heat under a changing climate. Only a handful of studies have examined healthcare costs associated with exposures to high temperatures. This research explores costs associated with hospitalizations for heat-related illness (HRI) in the United States using the 2001 to 2010 Nationwide Inpatient Sample (NIS). Descriptive statistics of patient data for HRI hospitalizations were examined and costs of hospitalizations were reported using the all-payer inpatient cost-to-charge ratio. Costs were examined using a log-gamma model with patient and hospital characteristics included as fixed effects. Adjusted mean costs were then compared across racial groups. The mean costs of HRI hospitalizations were higher among racial/ethnic minorities compared to Whites, who accounted for almost 65% of all HRI hospitalizations. Observed differences in costs based on income, insurance, and gender were also significant. These results suggest that these populations are suffering disproportionately from health inequity, thus, they could shoulder greater disease and financial burdens due to climate change. These findings may have important implications in understanding the economic impact public health planning and interventions will have on preventing hospitalizations related to extreme heat.

## 1. Introduction

Understanding how heat waves affect morbidity and mortality, as well as the associated economic costs, is essential for characterizing the human health impacts of extreme heat under a changing climate. Current research finds that mortality and morbidity increase during heat waves [1,2,3,4]. For example, Basu et al. (2012) observed a 393% increase in hospitalization for heat exposure, a 3% increase in ischemic stroke hospitalizations, and a 15% increase in acute renal failure hospitalizations in California for every 10°F increase above the mean ambient temperature [5]. As heat events increase, deaths and hospitalizations due to heat-related illnesses are expected to result in greater economic costs. This may be especially true for those living in poverty and some racial/ethnic minorities as current assessments of climate change effects on health are projected to disproportionately impact already disadvantaged populations [6,7,8]. Evidence also suggests that poor and racial/ethnic minority populations are often segregated in residential areas that are more vulnerable to heat-health risks, such as densely populated urban areas and areas with reduced vegetative cover (less green space), characterized as an “urban heat island” [9]. Additionally, populations that already have increased risk for heat-related illnesses, such as those with underlying medical conditions, including cardiac and respiratory diseases, and those without air conditioning or access to cooling centers, may suffer additional financial burdens from increased hospitalizations for heat-related illnesses due to additional medical costs and added household expenses [10]. 

Studies on the economic impacts of climate change have mainly focused on emissions of greenhouse gases (GHGs), particularly carbon dioxide, and the direct effects GHGs have on global warming and the economy, compared to direct health costs [11,12,13]. Though, more recently, studies have analyzed the economic costs of climate change on human health and livelihood, including the examination of mortality from climate change-related air quality impacts using the value of a statistical life (VSL) [14] and costs associated with lost labor due to temperature extremes [15,16]. While these and other studies on have estimated some of the direct and indirect economic costs, only a handful of studies have estimated costs associated with high ambient temperatures and extreme heat events [17,18,19]. Knowlton et al. (2011) identified total health costs associated with a 2006 California heat wave to be $5.4 billion, though a significant portion of the estimated cost was due to premature death based on a VSL approach ($5.1 billion) [17]. Another study by Lin et al. (2012) project that hospitalization costs for respiratory illnesses associated with high summer temperatures may increase from a baseline (1991–2004) of $0.64 million per year to $26–76 million per year by the end of the century [18].

This study aims to improve our understanding of the public health costs associated with high ambient temperatures by calculating the economic burden of hospitalizations due to heat-related illnesses in the United States from 2001 to 2010. This includes a targeted analysis of the differences across racial/ethnic and socioeconomic groups. The study findings are intended to inform future interventions aimed at reducing heat-related illness hospitalizations and the costs associated with climate impacts on the health and well-being of the most vulnerable populations including the poor and minorities.

## 2. Methods

### 2.1. Data Source and Study Population

The primary source of data for this study was the 2001 to 2010 Healthcare Cost and Utilization Project (HCUP) Nationwide Inpatient Sample (NIS), which was developed by the Agency for Healthcare Research and Quality (AHRQ). The NIS is the largest all-payer care database, with data from approximately 8 million hospitalizations per year (about 20% of all hospital discharges in the United States). The NIS includes data on patient demographics and hospital characteristics, primary and secondary diagnoses coded according to the *International Classification of Diseases, Ninth Revision* (ICD-9, up to 25 diagnoses), primary and secondary procedures, type and source of admission, discharge disposition, primary payer type, total hospital charges, and length of stay. Stratification and weighting variables allow calculation of national estimates and accounts for the complex sampling design [20,21].

The study population for HRI consists of patients in the NIS with at least one diagnosis of a heat-related illness (ICD-9 codes 992.0–992.9), which includes a spectrum of environment-related heat conditions, such as heat stroke (ICD-9 code 992.0), heat syncope (992.1), cramps (992.2), and edema (992.7), heat exhaustion to fatigue (992.3–992.6), and other unspecified heat effects (992.8–992.9) from 2001 to 2010. Only hospitalizations occurring during the summer months (May–September) were included in the analysis. All other hospital admissions during this time period were also examined and used for comparison.

The main outcome assessed in this study was total billed charges per hospitalization, which was assessed by mean adjusted costs. Total billed charges were converted to individual hospital all-payer inpatient costs by applying the AHRQ hospital-specific cost-to-charge ratios to total charges within the NIS dataset. Individual patient records were matched by hospital identification numbers and linked to AHRQ hospital-specific cost-to-charge ratios [22]. For hospitals without a listed all-payer inpatient cost-to-charge ratio a group average all payer-inpatient cost was used. Group average all payer-inpatient cost was substituted with a cost-to-charge ratio for hospitals in Texas, Pennsylvania and Hawai’i that did not have complete records of either form of cost-to-charge ratios. Costs for hospitals in the same census region were matched by location and bed size.

### 2.2. Data Analysis

Adjusted costs were calculated based on total-billed charges and applying the cost-to-charge ratios. Cost-to-charge ratios were supplied by the AHRQ and multiplied by the total-billed charges (Adjusted Costs = Total-Billed Charges * All-payer Inpatient Cost/Charge Ratio (APICC)). Adjusted hospitalization costs were then modeled using a log-gamma regression model to appropriately account for the non-normal distribution of costs [23,24]. Independent variables included patient characteristics (gender, age, race, zip-code income quartile, insurance type, and comorbidity index) and hospital characteristics (location, region, and bed size). Weighting variables were added to the model to produce national estimates. The log-gamma regression model used was hierarchical and included state and hospital as random effects to account for possible correlation within hospitals and states. The adjusted cost output from the log-gamma model was then averaged and aggregated by race, gender, insurance status, ages 65 and older, and zip-code income quartile. An ANOVA was used to examine differences in means for the cost of hospitalizations between populations, specifically race, insurance status, and zip-code income quartile. A referent group was identified and mean costs were compared against this group for each of the variables using Tukey’s HSD test. A *t*-test was used the compare differences in adjusted cost means for gender. All statistical analyses were performed using SAS 9.4 (SAS Institute, Cary, NC, USA) and the study conforms to the HCUP data use agreement.

## 3. Results

Table 1 and Table 2 show inpatient and hospital descriptive characteristics of all hospitalizations, as well as HRI hospitalizations during the study period. Patients with HRI tended to have a higher mean age, 55 years old, compared to a mean age of 47.7 years old for all other hospitalizations. There are also disproportionately fewer 0–17 year olds hospitalized for HRI, compared to all hospitalizations during this time period. Heat-related illness hospitalizations had shorter durations in length-of-stay and lower total charges than all hospitalizations. Heat-related illness hospitalizations resulted in 1353 (1.9%) deaths, with all hospitalizations having approximately the same percentage of deaths (2.0%) deaths during the study period. Additionally, patients with HRIs were more likely to be male (73.6%) compared to female (41.3%), reside in the lowest zip-code income quartile, 36.1% compared to 22.9%, not have insurance, 15.1% compared to 5.8%, and be hospitalized in rural areas, 23.8% compared to 12.1% for all other hospitalizations during this time period.

Apart from the descriptive statistics, there were also notable differences in the comparison of costs associated with HRIs and all other hospitalizations (Table 3). Using White as the reference group, costs for HRI hospitalized were greater among Blacks, Hispanics, and Asian/Pacific Islanders; only Blacks had elevated costs among all hospitalizations. Asian/Pacific Islanders showed the greatest differences with mean costs over $1,000 higher, per hospitalization, than Whites. Costs were also greater among Blacks for all hospitalizations (+$147 95% CI: $128, $166), with other race/ethnic groups having lower costs, though these costs were still greater than HRI-only hospitalization costs among racial/ethnic groups. These costs followed similar patterns between all hospitalization and HRI-only hospitalizations for zip-code income quartile, insurance status, ages 40+, and gender. Hospitalization costs among zip-code income quartiles increased as quartiles increased with the highest zip-code income quartile in all hospitalizations and HRI-only hospitalizations having the greater costs. Those who used Medicare/Medicaid and Other insurance types, including workers compensation and other state and federal insurance programs, had increased costs compared to patients with private insurance. Those aged 40 and over also had higher hospitalizations costs in general among both HRI and all other hospitalizations during this time period, compared to young adults 18–39 years old, though children, 0–17 years old, had lower hospitalization costs compared to the reference group. Differences in costs between genders was significant, with males having higher costs among all hospitalizations and females having higher costs among HRI hospitalizations.

Further examination of racial groups shows that among HRI patients with no insurance, hospitalization costs were higher among all racial/ethnic groups compared to patients identified as White. Among patients from the lowest zip-code income quartile, Blacks, Hispanics and Asian/Pacific Islanders had higher costs compared to Whites (Table 4). Similar patterns between genders were observed among racial groups with females identifying as Black, Hispanic and Asian/Pacific Islander having greater hospitalizations costs for HRIs than Whites (Table S1).

## 4. Discussion

Our analyses indicated that, compared to Whites, higher costs for heat-related hospitalizations were associated with some racial/ethnic minorities, particularly those residing in lower zip-code income areas and among the uninsured. Additionally, although accounting for a smaller portion of HRI hospitalizations, being female was associated with adjusted mean costs significantly higher than males. Patients using Medicaid/Medicare were also associated with significantly higher costs than those with private or HMO insurance, which could correlate to the number of poor and elderly that are hospitalized for HRIs.

While a prior study indicated that females were more likely than males to be hospitalized for heat-related respiratory illnesses [18], our study shows that among HRIs, females have fewer hospitalizations but bear a greater burden of the hospitalization costs. This could be due to different personal behaviors and working characteristics between genders during high temperature events [25]. Males may be more likely to have occupations that are outdoors, performing physical activity in high temperature environments, increasing their exposure. These males may be in generally good health (healthy worker effect) and may only suffer from minor heat-related illnesses, not incurring high healthcare costs. For females, we considered pregnancy as a potential cause for the increased costs among this population, though no pregnancy related diagnostic codes were associated with any discharges with a concurrent HRI in this study. More severe illnesses related to HRIs may have led to hospitalizations among females, which required treatments, not just for direct heat-related illnesses, but for additional diagnoses of underlying medical conditions. A recent study indicated that females were more likely to undergo a greater number of procedures and have a higher risk of non-routine discharges (e.g., transfers to a rehabilitation hospital) for hospitalizations associated with HRIs and respiratory diseases, which may result in higher healthcare costs [26]. While these characteristics may explain some of the differences in HRI hospitalization costs between genders, further research is needed to understand why females have higher costs though lower rates of HRI hospitalizations.

Our findings also indicate that adults aged 40–64 years old have a larger percentage of HRI hospitalizations during this time period, though older adults aged 65+ years old had significantly higher costs for HRI hospitalizations. Older adults were also more likely to be covered under Medicare. Patterns of hospitalization costs in the United States have been following this trajectory, with mean costs of all hospitalizations greatest among those aged 45–85 years old and for patients using Medicaid/Medicare, with average costs increasing by 0.9%–2.5% annually between 2003 and 2013 [27]. A study looking at hyperthermia-related inpatient and outpatient visits saw a doubling of visits among Medicare enrollees from 2004 to 2005 when average temperatures throughout the U.S. were 1.5°F higher than historical means, with the total cost of hyperthermia-related visits increasing from $11 to $25 million [28]. Underlying medical conditions prevalent in older adults, such as cardiac diseases and diabetes, may contribute to the high costs displayed within this age group as well as the increases we observed among those who used Medicare as their primary insurance. Additionally, if ambient temperatures continue to rise, as projected, more hospitalizations among older adults on Medicare may further economically strain an already taxed healthcare program [29]. Social isolation and lack of social engagement among older adults and the elderly are also predictors for health and may contribute to increased health disparities [30]. This in turn can contribute to the increased number of HRI hospitalizations among this population and added financial burdens. Increased education on the use of air conditioning, as we as outreach to older adults during heat events can help to reduce both the negative health effects and reduce the financial burden HRI hospitalizations will have on this population.

Research has shown that HRI hospitalizations have increased during our study period, with more recent climate data indicating that average global temperatures continued to rise during and after the 2001 to 2010 period [31,32]. Studies have already projected that there are likely to be increases in heat-related mortality, across the U.S., particularly under higher greenhouse gas emission scenarios [2,33]. Additionally, there is the potential for an increase in heat-related morbidity, due to extreme heat events, which may worsen health impacts and increase the financial burdens of individuals hospitalized for heat-related illnesses. Knowlton et al. (2011) calculated the health impact cost of a 2006 California heat wave event to be approximately $179 million U.S. dollars based on hospitalizations, emergency department visits, and outpatient visits [34]. While these costs are not broken down by any specific population group, such as race or gender, they provide an estimate of the financial impact heat events have on healthcare costs. Our findings suggest there are differences among certain vulnerable groups, which can be indicative of underlying vulnerabilities such as economic and healthcare inequalities.

When examining specific racial/ethnic hospitalizations, our results show that Blacks, Hispanics, and Asian/Pacific Islanders have higher hospital costs for HRIs than Whites. Racial/ethnic minorities are also at an increased risk for heat-related hospitalizations and mortality due to extreme heat events [10,34,35,36]. Race is a key determinant of health, in the United States, due to its association with poverty, discrimination and inequality in healthcare and healthcare access [37,38]. Inequalities, because of race/ethnicity and low socioeconomic status, can impose higher costs associated with the care of a sicker and more disadvantaged population [39]. This suggests that racial/ethnic minorities in the U.S. have greater health disparities, which in turn may impact the cost of hospitalization for these populations. Health and social inequities among low socioeconomic and minority populations can be attributed to a number of factors related to increased hospitalizations costs including, environmental justice issues, healthcare access and utilization, and lack of education [30]. A number of studies have also suggested that these marginalized groups face unequal exposures to climate hazards, referred to as the “climate gap” [40,41]. Our data lack the ability to answer these specific questions, though we have identified significant differences in HRI hospitalization costs among these marginalized populations, further research is needed to explore how issues of environmental justice, healthcare access, and health behaviors relate to HRI hospitalizations and increased hospital costs. Specific research questions should examine how environmental inequities, such as living in or near polluted areas, compound the risk of hospitalization associated with heat or other climate-related hazards among under-served populations.

Individuals with chronic medical conditions are also likely to incur greater costs associated with HRIs as certain conditions, such as respiratory or renal diseases, are known to be exacerbated by extreme heat. Preventative actions for reducing heat-related illness hospitalizations among the poor and minorities, such as using air conditioning, are limited, due to the impact of additional operating costs associated with having and using air conditioning or accessing cooling centers [36,42]. Efforts to promote health lifestyle and health awareness are often not targeted to the poor or other underserved populations and are more likely used by those with more resources and education to act on the information provided or to access services needed [30]. If public health interventions addressing heat-related morbidity and mortality are insufficient or ineffective, residents of poor and minority communities may need to rely on their own financial resources to address the economic and health inequities that they face when exposed to high ambient temperatures [6,43]. Ultimately, integrating what we know about health inequity and climate change planning can help to identify and implement strategies that address the disproportionate health and financial burdens faced by vulnerable populations. Towards this end, public health officials and policy makers will need to consider interventions accessible to low socioeconomic and minority communities. This may include health education programs among populations with English-language barriers or outreach among hard-to-reach populations, such as the homeless, both of whom may have limited access and means for healthcare.

Though the nationwide inpatient sample leverages a very large number of records over the study period, our study has several limitations. First, the NIS does not capture the specific date of admission, only day of the week and month, to protect patient confidentiality, so we could not link specific heat exposures to heat-related illnesses diagnoses. We limited the analyses to the summer months (May–September) as a proxy for exposure, which accounted for 95% of heat-related illness cases, though some could be unrelated to high ambient temperature exposure. Second, the NIS is an administrative database and the specificity and sensitivity of diagnosed cases are dependent on the coding. Administrative data is less accurate than clinical data and heat-related illnesses may often be under-reported. Although the NIS provides weights for national estimates, the 20% sample survey may be influenced by sampling error in which some populations may be over- or under-represented. Third, data from the NIS is also limited in its ability to track patients over time and after discharge. Patients diagnosed with severe heat-illnesses, like heat-stroke, may be transferred out of hospitals or discharged to rehabilitation facilities, which could incur additional costs not captured by this analysis. Additionally, patients that have less severe diagnoses of heat-illnesses may not be admitted to a hospital and only pass through emergency departments. This analysis only captures those patients that have been hospitalized and may not represent the total financial burden associated with heat-related morbidity and mortality.

## 5. Conclusions

The results of this study suggest healthcare costs for HRI hospitalizations are disproportionally higher for poor people as well as for some racial/ethnic minorities. Though while minority groups may not necessarily be labeled as poor, they still may incur higher HRI healthcare costs. In addition, those with Medicare/Medicaid and other insurance types also had higher HRI hospitalizations costs. These groups have been defined in the literature as being vulnerable to extreme heat and have an increased burden of disease from heat-related morbidity and mortality. Many factors such as underlying medical conditions, healthcare access and behaviors, and socioeconomic status contribute to these disparities and in turn may contribute to a greater economic costs associated with hospitalizations for heat-related illnesses. As climate change and average global temperatures continue to pose a threat to our health and well-being, strategic and cost-effective interventions should be identified and implemented, especially among underserved populations. Our findings reinforce the need for additional assessments of the financial impact that climate change, specifically high ambient temperatures, will have on population health and the healthcare system within the United States.

## Figures and Tables

**Table 1:** Sociodemographic characteristics of patients hospitalized with and without a heat-related illness (HRI), United States 2001–2010 (May–September).

Characteristics	All Hospitalizations	HRI Hospitalizations
N (total, weighted)	181,094,795	73,180
Age, Mean (±SD), y	47.7 (27.9)	55.0 (21.6)
Length of Stay, Median (IQR), d	3 (2–5)	2 (1–3)
Total Charges, Median (IQR), $	$12,480 ($5,956, $26,313)	$8,965 ($5,017, $17,047)
Death, n (%)	3,554,407 (2.0%)	1353 (1.9%)
Age Categories, %		
0–17	16.6%	4.4%
18–39	21.6%	20.5%
40–64	27.6%	38.2%
65–74	13.0%	13.4%
75+	21.3%	23.4%
Gender, %		
Male	41.2%	73.6%
Female	58.5%	26.4%
Missing	0.3%	-
Race/Ethnicity, %		
White	52.3%	64.8%
Black	11.2%	16.4%
Hispanic	10.4%	11.0%
Asian/Pacific Islander	1.8%	0.1%
Native American	0.4%	0.7%
Other	2.6%	2.6%
Missing	21.3%	4.3%
Zip-Code Income Quartile, %		
0 to 25th percentile	22.9%	36.1%
26th to 50th percentile	20.9%	25.9%
51st to 75th percentile	18.7%	18.8%
76th to 100th percentile	16.1%	13.8%
Missing	21.4%	5.3%
Payer–Primary, %		
Medicare/Medicaid	56.0%	48.9%
Private/HMO	34.8%	27.9%
None/Self-Pay	5.8%	15.1%
Other	3.2%	8.1%
Missing	0.2%	0.1%

IQR, interquartile range; SD, standard deviation.

**Table 2:** Hospital characteristics of patients hospitalized with and without a heat-related illness (HRI), United States 2001–2010 (May–September).

Characteristics	All Hospitalizations	HRI Hospitalizations
N (total, weighted)	181,094,795	73,180
Hospital Region, %		
Northeast	17.1%	11.6%
Mid-West	20.0%	18.8%
South	45.6%	54.4%
West	17.3%	15.2%
Hospital Size (# of beds), %		
Small	11.9%	17.1%
Medium	24.5%	26.6%
Large	63.3%	55.8%
Missing	0.3%	0.5%
Hospital Location, %		
Rural	12.1%	23.8%
Urban	87.6%	75.8%
Missing	0.3%	0.4%

**Table 3:** Comparisons of nationally adjusted mean costs per hospitalization by race, zip-code income quartile, insurance type, age 65 and older, and gender, 2001–2010 (May–September).

Characteristic	All Hospitalizations	HRI Hospitalizations
	Adjusted Mean Cost, 95% CI (U.S. Dollars)	Adjusted Mean Cost, 95% CI (U.S. Dollars)
Overall Costs	$8,836 ($8,831, $8,840)	$5,359 ($5,327, $5,390)
Race		
White	ref. ^1^	ref. ^2^
Black	$147 ($128, $166)	$319 ($197, $440)
Hispanic	−$1,377 (−$1,396, −$1,358)	$243 ($98, $387)
API	−$747 (−$790, −$704)	$1,208 ($793, $1,624)
Native American	−$634 (−$723, −$544)	−$596 (−$1,130, −$62)
Other	−$618 (−$655, −$581)	−$467 (−$755, −$178)
Zip-Code Income Quartile		
0 to 25th percentile	−$809 (−$826, −$793)	−$814 (−$935, −$692)
26th to 50th percentile	−$764 (−$782, −$747)	−$531 (−$660, −$402)
51st to 75th percentile	−$484 (−$501, −$466)	−$475 (−$612, −$338)
76th to 100th percentile	ref. ^1^	ref. ^2^
Insurance Type		
Private/HMO	ref. ^1^	ref. ^2^
None/Self-Pay	−$571 (−$597, −$546)	−$206 (−$319, −$93)
Other	$1,901 ($1,868, $1,935)	$460 ($315, $606)
Age Group		
0–17	−$1,356 (−$1,368, −$1,344)	−$411 (−$621, −$201)
18–39	ref. ^1^	ref. ^2^
40–64	$4,867 ($4,856, $4,877)	$1,128 ($1,020, $1,236)
65–74	$6,090 ($6,077, $6,103)	$1,547 ($1,409, $1,687)
75+	$4,554 ($4,543, $4,566)	$1,586 ($1,466, $1,707)
Gender		
Male	$10,225 ($10,419, $10,233)	$5,168 ($5,133, $5,203)
Female	$7,855 ($7,850, $7,860)	$5,922 ($5,858, $5,985)

ANOVA comparisons were significant at *p* < 0.05 using Tukey’s HSD and TTest for Gender was significant at *p* < 0.01. ^1^ Adjusted mean costs for All Hospitalization referent groups (95% CI): White, $9,040 ($9,034, $9,045); 76th to 100th percentile, $9,385 ($9,375, $9,395); Private/HMO, $7,855 ($7,848, $7,862); 18–39 year olds, $5,926 ($5,920, $5,931). ^2^ Adjusted mean costs for HRI Hospitalization referent groups (95% CI): White, $5,279 ($5,242, $5,316); 76th to 100th percentile, $5,904 ($5,826, $5,983); Private/HMO, $4,460 ($4,587, $4,692); 18–39 year olds, $4,369 ($4,306, $4,432).

**Table 4:** Comparison of nationally adjusted mean costs per hospitalization for patients with no insurance and patients in the lowest zip-code income quartile, stratified by race 2001–2010 (May–September).

Characteristic	All Hospitalizations	HRI Hospitalizations
	Adjusted Mean Cost, 95% CI (U.S. Dollars)	Adjusted Mean Cost, 95% CI (U.S. Dollars)
No Insurance, by Race		
White	ref. ^1^	ref. ^2^
Black	$747 ($688, $797) ^3^	$432 ($245, $620) ^3^
Hispanic	−$670 (−$723, −$617) ^3^	$262 ($71, $453) ^3^
API	−$305 (−$458, −$153) ^3^	$367 (−$607, $1,341)
Native American	−$97 (−$371, $176)	−$990 (−$1,859, −$120) ^3^
Other	$187 ($87, $284) ^3^	−$309 (−$736, $117)
Lowest Zip-Code Income Quartile, by Race		
White	ref. ^1^	ref. ^2^
Black	$658 ($628, $687) ^3^	$733 ($548, $918) ^3^
Hispanic	−$871 (−$903, −$839) ^3^	$703 ($472, $934) ^3^
API	−$41 (−$155, $76)	$1,238 ($341, $2,136) ^3^
Native American	−$470 (−$600, −$339) ^3^	−$68 (−$752, $615)
Other	−$361 (−$434, −$289) ^3^	−$335 (−$817, $147)

^1^ Adjusted mean costs for All Hospitalization referent groups (95% CI): No Insurance by Race–White, $7,279 ($7,259, $7,299); Lowest Zip-Code Income Quartile by Race White, $8,589 ($8,578, $8,601); ^2^ Adjusted mean costs for HRI Hospitalization referent groups (95% CI): No Insurance by Race–White, $4,304 ($4,236, $4,327); Lowest Zip-Code Income Quartile by Race White, $4,812 ($4,741, $4,882); ^3^ Comparisons significant at the *p* < 0.05 level using Tukey’s HSD.

## Supplementary Materials

The following are available online at www.mdpi.com/1660-4601/13/9/894/s1, Table S1: Comparison of nationally adjusted mean costs per hospitalization for patients by race, stratified by gender 2001–2010 (May–September).
