# Supplementary material for: Economic Burden of Hospitalizations for Heat-Related Illnesses in the United States, 2001–2010
Source: Int J Environ Res Public Health. 2016 Sep 8;13(9):894. doi: 10.3390/ijerph13090894 (PMC5036727; doi:10.3390/ijerph13090894)
Supplement: Supplementary file 1 [file ijerph-13-00894-s001.pdf]

Supplementary Materials: Economic Burden of Hospitalizations for Heat-Related Illnesses in the United States, 2001–2010

Michael T. Schmeltz, Elisaveta P. Petkova and Janet L. Gamble

Table S1. Comparison of nationally adjusted mean costs per hospitalization for patients by race, stratified by gender 2001–2010 (May–September).

Characteristic		All Hospitalizations Adjusted Mean Cost, 95% CI (US Dollars)	HRI Hospitalizations Adjusted Mean Cost, 95% CI (US Dollars)
Race, by Gender			
White	M	$10,428 ($10,419, $10,437)	$5,101 ($5,060, $5,143)
	F	$8,020 ($8,014, $8,025)	$5,777 ($5,703, $5,851)
Black	M	$10,600 ($10,577, $10,623)	$5,379 ($5,292, $5,466)
	F	$8,237 ($8,223, $8,251)	$6,241 ($6,092, $6,391)
Hispanic	M	$8,899 ($8,877, $8,922)	$5,328 ($5,228, $5,428)
	F	$6,887 ($6,875, $6,899)	$6,340 ($6,112, $6,567)
API	M	$9,746 ($9,687, $9,805)	$6,147 ($5,672, $6,623)
	F	$7,405 ($7,374, $7,437)	$7,010 ($6,484, $7,536)
Native American	M	$9,753 ($9,636, $9,871)	$4,477 ($4,019, $4,936)
	F	$7,475 ($7,400, $7,550)	$5,444 ($4,475, $6,413)
Other	M	$9,851 ($9,803, $9,899)	$4,581 ($4,388, $4,774)
	F	$7,412 ($7,383, $7,440)	$5,627 ($5,203, $6,050)

All *t*-test comparisons were significant at the $p < 0.01$ level.

© 2016 by the authors; licensee MDPI, Basel, Switzerland. This article is an open access article distributed under the terms and conditions of the Creative Commons by Attribution (CC-BY) license (http://creativecommons.org/licenses/by/4.0/).
